# Retinal Oximetry in a Healthy Japanese Population

**DOI:** 10.1371/journal.pone.0159650

**Published:** 2016-07-19

**Authors:** Yuki Nakano, Takeru Shimazaki, Nobuko Kobayashi, Yukiko Miyoshi, Aoi Ono, Mamoru Kobayashi, Chieko Shiragami, Kazuyuki Hirooka, Akitaka Tsujikawa

**Affiliations:** Department of Ophthalmology, Kagawa University Faculty of Medicine, Miki, Japan; Bascom Palmer Eye Institute, University of Miami School of Medicine;, UNITED STATES

## Abstract

**Purpose:**

To establish the normative database of retinal oximetry using Oxymap T1 in a healthy Japanese population, and study the reproducibility of the measurements in Japanese.

**Methods:**

We measured oxygen saturation in the major retinal vessels with Oxymap T1 in 252 eyes of 252 healthy Japanese subjects. Fundus images acquired using Oxymap T1 were processed using built-in Oxymap Analyzer software. Reproducibility of retinal oximetry was investigated using 20 eyes of 20 healthy subjects.

**Results:**

The mean retinal oxygen saturation of 4 quadrants in healthy Japanese was 97.0 ± 6.9% in arteries and 52.8 ± 8.3% in veins. The mean arteriovenous difference in oxygen saturation was 44.2 ± 9.2%. Both arterial and venous oxygen saturation were significantly lower in the temporal side of the retina, especially in the temporal-inferior vessels. However, the arteriovenous difference in oxygen saturation was limited in the 4 quadrants. Interphotograph, intervisit, and interevaluator intraclass correlation coefficients were 0.936–0.979, 0.809–0.837, and 0.732–0.947, respectively. In the major retinal arteries, oxygen saturation increased with age (r = 0.18, p<0.01), at a rate of 0.67% per 10 years. However, venous oxygen saturation showed no correlation with age.

**Conclusions:**

This study provides the normative database for the Japanese population. The arterial saturation value appears to be higher than other previous studies. Mean retinal oximetry in 4 quadrants with Oxymap T1 has high reproducibility.

## Introduction

Oxygen supply is essential for physiological functioning and metabolism in human organs. Retinal neurons need more oxygen than other tissues [1], and abnormal retinal oxygenation is thought to play a pivotal role in the pathogenesis of various retinal diseases such as diabetic retinopathy, retinopathy of prematurity, and retinal vein or artery occlusion [2,3]. *In vivo* measurement of retinal oxygen levels would be of help for the management of such diseases [4]. Recently, a non-invasive retinal oximeter Oxymap T1 has become commercially available. Oxymap T1 is based on the principle that deoxyhemoglobin and oxyhemoglobin have different light absorbance at specific wavelengths of light (570 nm and 600 nm) [5].

Using Oxymap T1, various investigators have reported the oxygen levels in the major retinal vessels and studied the repeatability of the measurements in healthy populations [6,7], mainly in Caucasians [8–11]. In addition, recent studies have reported the retinal oxygen concentration in eyes with retinal pigmentosa [12,13], glaucoma [7,14–19], high myopia [20], or diabetic retinopathy [4,21]. Longitudinal studies on retinal oximetry would contribute to the early detection and management of such chronic diseases. In diabetic patients, the level of oxygen saturation is reported to be increased in the major retinal veins, as early as the stage of background retinopathy or even no retinopathy [21]. In eyes with retinitis pigmentosa, the retinal venous oxygen saturation is correlated with the residual visual field [18].

However, previous studies have indicated that retinal oximetry may be influenced by the flash intensity [22], face position [11], or the degree of fundus pigmentation [23,24]. So far, limited information is available on either the normative data for the Japanese population [18] or the reproducibility of retinal oximetry in the Japanese. In addition, only one previous report investigated the intervisit reproducibility of Oxymap T1 [10], which is essential to set up and interpret in longitudinal studies.

Thus, the purpose of the current study was to establish a normative database for retinal oximetry using Oxymap T1 in a healthy Japanese population, and study the reproducibility of the measurements in Japanese.

## Patients and Methods

The Ethics Committee at Kagawa University Faculty of Medicine approved this prospective study, which was conducted in accordance with the tenets of the Declaration of Helsinki. Written informed consent was obtained from each subject before any study procedures or examinations were performed. This study is registered in ClinicalTrials.gov (NCT02318641).

### Subjects

This prospective study consisted of 252 eyes with no ocular disease of 252 healthy Japanese subjects. Oxygen saturation in the major retinal vessels was studied using Oxymap T1 (Oxymap ehf, Reykjavik, Iceland) at the Kagawa University Hospital Department of Ophthalmology between December 2014 and June 2015. Exclusion criteria were as follows: keratoconus, high myopia (more severe than -6 diopters), high astigmatism (more severe than ± 3 diopters), best-corrected visual acuity worse than 20/25, prior intraocular surgery, or any co-existing ocular disease (e.g., glaucoma, diabetic retinopathy, retinal vein or artery occlusion, hypertension retinopathy, age-related macular degeneration, retinal degenerative disease, or senile cataract that diminished image quality). Patients who had suspected adverse effects to pupil dilation, diabetes mellitus, severe cardiovascular or respiratory diseases, pregnancy, or collagen disease were also excluded from the current study.

### Image acquisition for retinal oximetry

**Fig 1:**
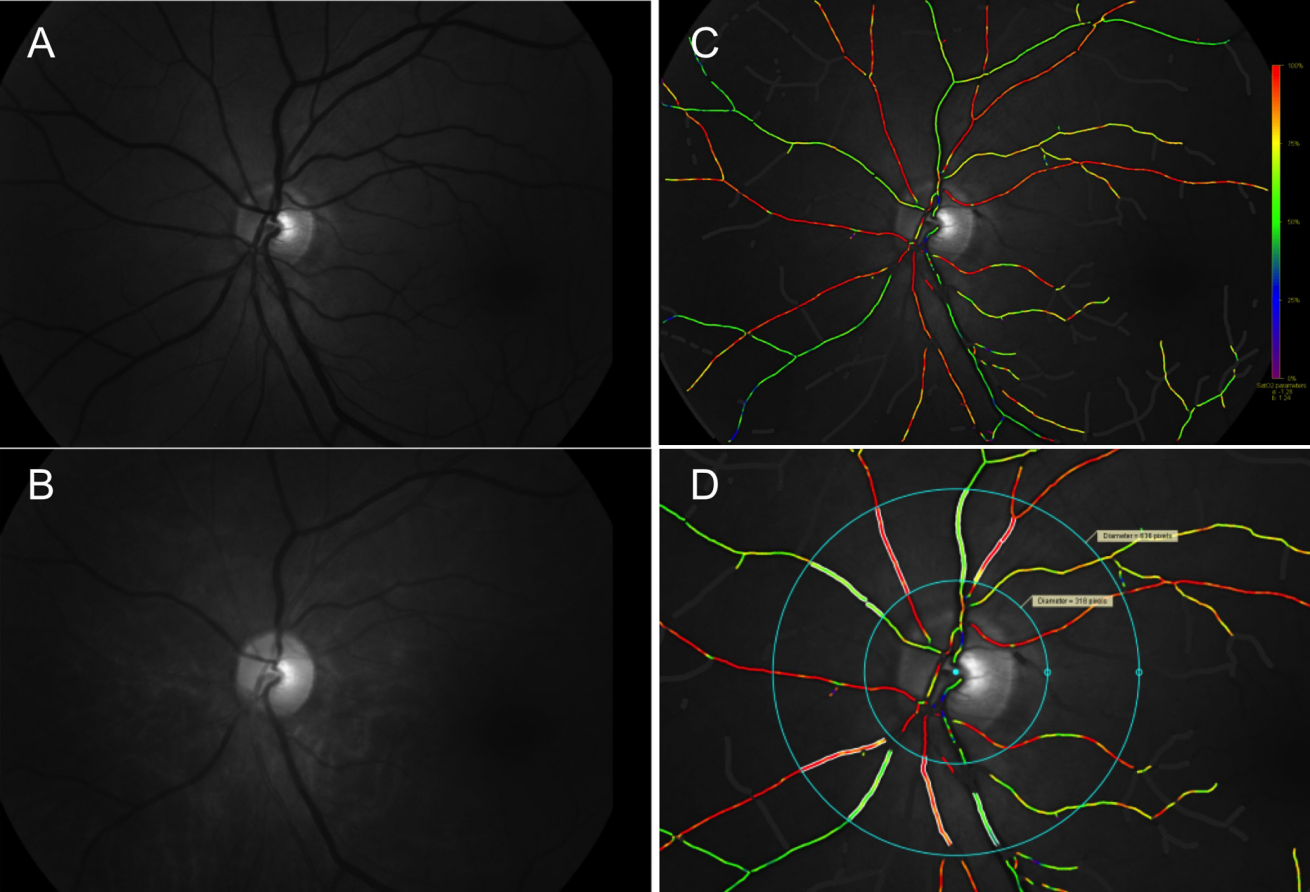
Fundus images at two different wavelengths of light obtained by Oxymap T1 and the oxygen saturation map analyzed by Oxymap Analyzer. (**A**, **B**) Oxymap T1 simultaneously captures 50° monochrome images centered at the optic disc at two different wavelengths of light (570 nm and 600 nm). (**A**) 570 nm is the reference isosbestic wavelength that is insensitive to oxygen concentration. (**B**) 600 nm is the oxygen-sensitive wavelength. (**C**) Oxymap Analyzer automatically calculates the oxygen saturation in each selected vessel and produces a color oxygen saturation map. Retinal vessels with higher oxygen saturation are indicated with red to orange color. Green to purple color indicates the retinal vessels with lower oxygen saturation. (**D**) For the analysis, vessel selection was performed disc-centered, 1.5-to 3-disc diameters area and 1 disc diameter away from the disc margin.

The principle of a commercially available retinal oximeter Oxymap T1 has been described previously by other researchers [4]. In brief, Oxymap T1 is composed of two digital cameras, an image splitter, and two narrow band-pass filters and is attached to a fundus camera (TRC-50DX, Topcon, Tokyo, Japan). Oxymap T1 simultaneously captures two fundus images at two different wavelengths of light (570 nm and 600 nm) (Fig 1). Light of 570 nm, which is isosorbetic to deoxyhemoglobin and oxyhemoglobin, is insensitive to oxygen saturation, and light of 600 nm, which is more sorbetic to oxyhemoglobin than deoxyhemoglobin, is sensitive to oxygen saturation.

After a comprehensive medical interview, all subjects underwent a thorough ocular examination, including autorefractometer, best-corrected visual acuity measurement with a 5-m Landolt chart, slit lamp examination, intraocular pressure measurement, dilated funduscopy, 50° digital fundus photographs, and optical coherence tomography (Spectralis HRA+OCT; Heidelberg Engineering, Heidelberg, Germany). When both eyes met the inclusion criteria, one eye was selected randomly for the examination.

After mydriasis with 0.5% of tropicamide and 0.5% of phenylephrine hydrochloride (Mydrin-P; Santen, Osaka, Japan), each included eye was examined with Oxymap T1 (Fig 1) in a dark room. Setting of Oxymap T1 for image acquisition was as follows: flash intensity was 50 W and small aperture and large pupil settings were applied to the fundus camera. Fifty-degree fundus images were obtained centered at the optic disc by using Oxymap T1. At each setting, more than 2 images were obtained per eye.

### Oxygen saturation and vessel diameter measurement from acquired images

Fundus images acquired with Oxymap T1 were processed using a built-in software Oxymap Analyzer (version 2.4.2, Oxymap Ehf) [4]. The software calculates optical density of retinal vessels at two wavelengths (570 nm and 600 nm) (Fig 1). The ratio of the optical densities at these two wavelengths has an inverse and approximately linear relationship with oxygen saturation. The resolution of Oxymap T1 is 9 μm [25].

In each acquired image centered at the optic disc, the oxygen saturation was calculated in major retinal arteries and veins measuring more than 6 pixels in vessel width in the measurement zone. The evaluators set analyzed vessel segments in a standardized manner. For the analysis, vessel selection was done with the disc centered in a 1.5-to 3-disc diameter area and 1 disc diameter away from the disc margin to avoid uneven retinal background reflections near the optic disc margin (Fig 1) [23]. All branches and vessel crossings within the measurement area were also manually excluded from the analysis. After detailed selection of the vessel section for the analysis, Oxymap Analyzer automatically measured the levels of oxygen saturation and vessel diameter in each selected vessel.

### Reproducibility of retinal oximetry with Oxymap T1

Reproducibility of retinal oximetry was examined in 20 eyes of 20 healthy subjects (15 men and 5 women; 5 right and 15 left eyes; mean age, 24.0 ± 1.7 years [range, 23 to 29 years]). To investigate interphotograph reproducibility of retinal oximetry measurements, an evaluator (YN) performed the calculation of the levels of oxygen saturation in each major retinal vessel with Oxymap Analyzer, from two different photographs obtained by Oxymap T1 on the same day. To investigate intervisit reproducibility of retinal oximetry measurements, 2 photographs were obtained by the same photographer at 2 different visits. Photography was performed 1 week apart. Calculation of the levels of oxygen saturation in each major retinal vessel was performed by the same evaluator (YN). To investigate interevaluator reproducibility of retinal oximetry measurements, two different evaluators (TS, YN) independently performed calculation of the levels of oxygen saturation in each major retinal vessel from a single photograph. The intraclass correlation coefficient (ICC) was used to determine the reproducibility using oxygen saturation in a major retinal vessel in each quadrant and the mean of all 4 major retinal arteries and veins.

### Statistical analysis

All data were collected in an Excel database (Microsoft Office 2010; Microsoft, Redmond, WA). All statistical analyses were conducted using Software R (version 2.8.1; R Foundation for Statistical Computing, Vienna, Austria). The oxygen saturation was calculated in each major retinal artery and vein in 4 quadrants, the mean of values of 2 vessels in superior or inferior hemispheres, and the mean of values of 4 vessels in all quadrants. Pearson’s correlation coefficient was calculated to assess the relationship between age, refractive sphere, or vessel diameter and the level of oxygen saturation. Stepwise forward multivariate linear regression analyses were also performed to evaluate the contribution that each initially identifiable factor made to retinal oximetry. A p value <0.05 was considered to be statistically significant.

## Results

In the current study, retinal oximetry was performed in 252 eyes of 252 healthy Japanese subjects (137 men and 115 women). Mean age was 61.1 ± 18.8 years and ranged between 20 and 93 years (Table 1). Oxymap T1 allowed us to capture the monochromatic fundus images at two wavelengths, and Oxymap Analyzer calculated the oxygen saturation in each major retinal vessel. Mean oxygen saturation in 4 quadrants was 97.0 ± 6.9% in arteries and 52.8 ± 8.3% in veins. The mean arteriovenous (A-V) difference in mean oxygen saturation was 44.2 ± 9.2%. Table 2 shows the oxygen saturation in each major retinal artery and vein in 4 quadrants, the mean of values of 2 vessels in each hemisphere, and the mean of values of 4 vessels in all quadrants. Measurements of both arterial and venous oxygen saturation were significantly lower in the temporal side of the retina, especially in the temporal-inferior vessels. However, there were limited differences in A-V difference of oxygen saturation in the 4 quadrants (Table 2). Table 3 shows the mean oxygen saturation and mean vessel diameter in major retinal vessels in all groups stratified by age.

**Table 1:** Systemic and Ocular Characteristics of Healthy Japanese Recruited in the Current study.

Age, years, (mean ± standard deviation, range)	61.1 ± 18.8 (20 to 93)
20–39 years, number of eyes	40
40–59 years, number of eyes	45
60–79 years, number of eyes	134
80+ years, number of eyes	33
Total, number of eyes	252
Gender, male/female	137/115
Refractive sphere, diopter, (mean ± standard deviation, range)	-1.08 ± 2.92 (-6 to +4)

**Table 2:** Oxygen Saturation in Major Retinal Vessels in a Healthy Japanese Population Measured by Oxymap T1.

**Overall**	**Artery, %**	**Vein, %**	**A-V difference, %**
Temporal-superior vessel	95.7 ± 8.8**	52.1 ± 9.3**	43.6 ± 11.0*
Temporal-inferior vessel	92.7 ± 10.4	47.5 ± 10.6	45.4 ± 13.0
Nasal-superior vessel	100.7 ± 9.6**	55.9 ± 9.2**	44.8 ± 11.3
Nasal-inferior vessel	99.1 ± 10.5**	55.0 ± 10.6**	44.1 ± 13.8
Mean of 2 temporal vessels	94.1 ± 8.1**	49.9 ± 8.9**	44.3 ± 10.1**
Mean of 2 nasal vessels	99.8 ± 8.2**	55.4 ± 8.8**	44.4 ± 10.5
Mean of 2 superior vessels	98.0 ± 7.8**	54.1 ± 8.3**	44.0 ± 9.7
Mean of 2 inferior vessels	95.9 ± 8.6**	51.4 ± 9.6**	44.5 ± 11.2
Mean of 4 vessels	97.1 ± 6.9**	52.8 ± 8.3**	44.2 ± 9.2

A-V, arteriovenous.

*p<0.05

**p<0.01, compared with values in measurement values of temporal-inferior vessel (Wilcoxon signed-rank test).

**Table 3:** Mean Oxygen Saturation and Mean Vessel Diameters in Major Retinal Vessels in a Healthy Japanese Population Measured using Oxymap T1.

**Mean of oxygen saturation**	**Artery, %**	**Vein, %**	**A-V difference, %**
Overall	97.1 ± 6.9	52.8 ± 8.3	44.2 ± 9.2
Age 20–39	93.9 ± 5.4	53.5 ± 6.0	40.4 ± 7.2
Age 40–59	97.8 ± 6.9	54.4 ± 7.8	43.4 ± 9.3
Age 60–79	97.4 ± 7.2	51.8 ± 9.0	45.6 ± 9.5
Age 80+	98.0 ± 6.6	56.7 ± 8.2	44.3 ± 8.8
**Mean of vessel diameter**	**Artery,** μ**m**	**Vein,** μ**m**	
Overall	122.6 ± 14.0	164.8 ± 15.4	-
Age 20–39	120.8 ± 11.7	158.9 ± 14.6	-
Age 40–59	119.7 ± 13.8	163.2 ± 17.2	-
Age 60–79	124.6 ± 14.4	167.3 ± 14.6	-
Age 80+	120.5 ± 14.1	163.9 ± 15.2	-

Reproducibility of retina oximetry was examined in 20 eyes of 20 healthy subjects. Table 4 shows the ICCs in arterial and venous oximetry and A-V differences. Interphotograph, intervisit, and interevaluator ICCs of the retinal oximetry in one vessel were 0.891–0.970, 0.766–0.949, and 0.379–0.922, respectively. Although interphotograph ICC was high, interevaluator ICC was relatively low. However, the mean retinal oximetry in 4 quadrants had high ICCs between two photographs (0.936–0.979), two visits (0.809–0.837), or two evaluators (0.732–0.947).

**Table 4:** Intraclass Correlation Coefficients for Oxygen Saturation in Major Retinal Vessels Measured by Oxymap T1.

	**Intraclass Correlation Coefficient (95% CI)**		
	**Interphotograph**	**Intervisit**	**Interevaluator**
Arterial saturation			
Mean of 4 vessels	0.979 (0.948–0.992)	0.810 (0.528–0.924)	0.947 (0.864–0.979)
Temporal-superior vessels	0.944 (0.861–0.978)	0.878 (0.697–0.951)	0.857 (0.639–0.943)
Temporal-inferior vessels	0.915 (0.789–0.966)	0.858 (0.348–0.943)	0.888 (0.742–0.954)
Nasal-superior vessels	0.951 (0.880–0.981)	0.785 (0.457–0.914)	0.711 (0.297–0.884)
Nasal-inferior vessels	0.960 (0.900–0.984)	0.846 (0.618–0.939)	0.918 (0.793–0.968)
Venous saturation			
Mean of 4 vessels	0.954 (0.886–0.982)	0.837 (0.596–0.935)	0.884 (0.713–0.912)
Temporal-superior vessels	0.970 (0.925–0.988)	0.949 (0.873–0.980)	0.710 (0.268–0.885)
Temporal-inferior vessels	0.927 (0.819–0.971)	0.846 (0.617–0.939)	0.626 (0.098–0.849)
Nasal-superior vessels	0.975 (0.938–0.990)	0.911 (0.978–0.965)	0.918 (0.9795–0.968)
Nasal-inferior vessels	0.912 (0.974–0.965)	0.766 (0.429–0.907)	0.922 (0.803–0.969)
Arteriovenous difference			
Mean of 4 vessels	0.936 (0.841–0.974)	0.809 (0.526–0.924)	0.732 (0.345–0.892)
Temporal-superior vessels	0.909 (0.775–0.964)	0.810 (0.529–0.924)	0.379 (0.202–0.872)
Temporal-inferior vessels	0.936 (0.842–0.975)	0.807 (0.522–0.923)	0.600 (0.050–0.837)
Nasal-superior vessels	0.891 (0.730–0.957)	0.795 (0.491–0.918)	0.484 (-0.241–0.791)
Nasal-inferior vessels	0.926 (0.817–0.971)	0.849 (0.626–0.940)	0.901 (0.749–0.961)

**Fig 2:**
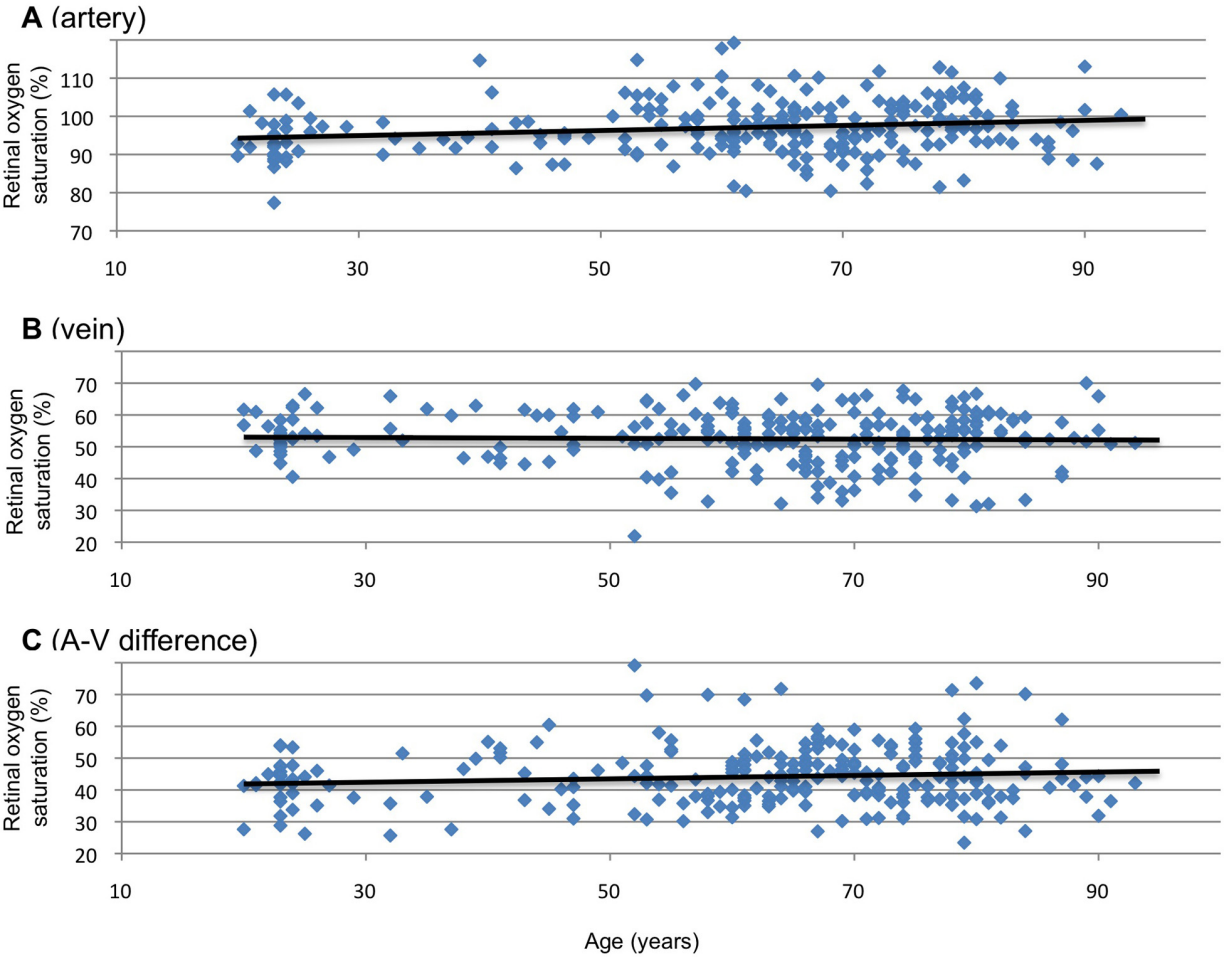
Association between retinal oxygen saturation and age in healthy Japanese. (**A**) Mean oxygen saturation in major retinal arteries in 4 quadrants (r = 0.18, p<0.01). (**B**) Mean oxygen saturation in major retinal veins in 4 quadrants (r = -0.06). (**C**) Mean arteriovenous (A-V) difference in mean oxygen saturation in 4 quadrants (r = 0.19, p<0.01).

Fig 2 and Table 5 show the associations between the retinal oxygen saturation and age in healthy subjects. In the major retinal arteries, oxygen saturation increased with age (r = 0.18, p<0.01). Arterial oxygen saturation increased by 0.67% per 10 years. However, the venous oxygen saturation showed no correlation with age (r = -0.06). A-V difference in retinal oxygen saturation increased with age (r = 0.19, p<0.01) by 0.92% per 10 years.

**Table 5:** Association between Age and Oxygen Saturation in Major Retinal Vessels Measured by Oxymap T1.

	**Artery**	**Vein**	**A-V difference**
Temporal-superior vessel	0.26**	0.03	0.18**
Temporal-inferior vessel	0.10	-0.07	0.14*
Nasal-superior vessel	0.13*	-0.06	0.16**
Nasal-inferior vessel	0.07	-0.04	0.09
Mean of 2 temporal vessels	0.20**	-0.04	0.19**
Mean of 2 nasal vessels	0.13*	-0.06	0.15*
Mean of 2 superior vessels	0.20**	-0.03	0.19**
Mean of 2 inferior vessels	0.08	-0.06	0.12
Mean of 4 vessels	0.18**	-0.06	0.19**

A-V, arteriovenous.

Pearson’s correlation coefficient was used.

*p<0.05

**p<0.01.

Table 6 shows the correlation between refractive sphere, age, or vessel diameter and oxygen saturation. There was no association between refractive sphere and oxygen saturation in the major retinal vessels. Multivariate analysis showed that arterial retinal saturation was significantly associated with age (β = 0.026, p = 0.002) and vessel diameter (β = -0.16, p = 0.013). The A-V difference was significantly associated with age (β = 0.19, p = 0.003).

**Table 6:** Correlation between Refractive Sphere, Age, or Vessel Diameter, and Oxygen Saturation in the Major Retinal Vessels.

	*Pearson’s correlation*		*Stepwise forward regression*	
	**r**	**p-value**	**Standardized β**	**p-value**
**Artery**				
Refractive sphere	0.01	0.934	-	-
Age	0.18	0.004	0.026	0.002
Vessel diameter	-0.15	0.021	-0.16	0.013
**Vein**				
Refractive sphere	-0.05	0.436	-	-
Age	-0.06	0.366	-	-
Vessel diameter	-0.02	0.702	-	-
**A-V difference**				
Refractive sphere	0.05	0.435	-	-
Age	0.19	0.003	0.19	0.003

Adjusted R^2^ (the coefficient of multiple determination) = 0.049 (artery), 0.032 (A-V difference).

r = Pearson’s correlation coefficient; β = regression coefficient.

## Discussion

In the current study, retinal oximetry was performed in 252 eyes of 252 healthy Japanese subjects, which is the largest normative database of Oxymap T1 to date. In a study on 120 Caucasians, Geirsdooier et al.[26] reported that retinal oxygen saturation was 92.2 ± 3.7% in arterioles and 55.6 ± 6.3% in venules, and the A-V difference was 36.7 ± 5.4%. In a study on 118 Asians, Yip et al.[6] reported that the oxygen saturation was 93.64 ± 6.9% in retinal arterioles and 54.22 ± 6.9% in venules, and the A-V difference was 39.43 ± 8.9%. In a recent study on 98 Indians, Mohan et al.[27] reported that the oxygen saturation was 90.3 ± 6.6% in retinal arterioles and 56.9 ± 6.3% in venules, and the A-V difference was 33.2 ± 5.2%. Thus, it is important to note that the results of retinal oximetry with Oxymap T1 show variation across races, and this fact should be kept in mind while obtaining and interpreting measurements.

We used the default formula of Oxymap Analyzer to calculate the retinal oxygen concentration [4], which uses the mean oxygen saturation values obtained from retinal vessels in healthy individuals by Schweitzer et al.[28]. In their measurement, the mean oxygen saturation was 92.2 ± 4.1% in retinal arteries and 57.9 ± 9.9% in retinal veins [28]. In a recent study of Oxymap T1 by Ueda-Consolve et al.[26], the mean arterial and venous oxygen saturation values in 14 healthy Japanese were 99.9 ± 8.9% and 54.6 ± 6.3%, respectively. We can estimate that the A-V difference was 45.3%, which is relatively higher than that reported in previous reports, which were mainly on Caucasians. Two fundus images captured at two different wavelengths of light by Oxymap T1 may be influenced by the degree of fundus pigmentation [23,24]. Because the fundus of Japanese subjects is more pigmented than that of Caucasians, this difference may account for the racial variations in the measurements of retinal oximetry. We may need to establish a calibration formula optimized for each race.

In the current study, measurements of both arterial and venous oxygen saturation were significantly lower in the temporal hemisphere, especially in the temporal-inferior quadrant [29]. Geirsdooier et al.[26], Mohan et al.[27], and Palsson et al.[11] reported similar findings. The exact reason for this regional variation is unclear. Indeed, venous oxygen concentration may be influenced by retinal metabolism. However, arterial oxygen concentration should not show such a great variation because all retinal arteries originate from a single central retinal artery. Rather, this variation may be explained by the measurement error, perhaps on the photographs by Oxymap T1 [22], because venous oxygen saturation was also lower in the temporal hemisphere and the A-V difference was almost canceled out. Recently, Mohan et al.[30] reported a strong negative correlation between calculated oxygen saturations measured using Oxymap T1 and peripapillar retinal nerve fiber thickness, which is large in the temporal hemisphere. This finding would support our current finding that both arterial and venous oxygen saturation were low in the temporal hemisphere. We measured oxygen saturation in all 4 quadrants. To minimize the error in photography, it would be most appropriate to calculate the mean of the values in all quadrants.

In the current study on a healthy Japanese population, interphotograph, intervisit, and interevaluator ICCs for retinal oximetry in one vessel ranged from 0.891 to 0.970, from 0.766 to 0.949, and from 0.379 to 0.922, respectively. Interphotograph ICC in our measurement was high and Palsson et al.[11], Goharian et al.[7], and Yip et al.[6] also reported high interphotograph (intravisit) reproducibility. In addition, O'Connell et al.[10] reported relatively high intervisit reproducibility of retinal oximetry with Oxymap T1. However, it is suggested that pupil size or face position may influence the measurements in retinal oximetry [11]. This would account for the slightly lower intervisit ICC in the current population, compared with interphotograph ICC. In the current study, interevaluator ICC was lower than interphotograph or interevaluator ICCs. In a study by Yip et al.[6], intergrader (interevaluator) ICC was 0.77–0.94. Although Oxymap Analyzer has a built-in, semiautomatic software to calculate the oxygen concentration, the selection of the vessel segment depends on the evaluator [4]. In addition to the standardization of retinal image capture by Oxymap T1 [11], the standardization of analysis with Oxymap Analyzer is also essential to maximize the reproducibility of retinal oximetry [6].

In our subjects, while arterial oxygen saturation increased with age, venous oxygen saturation showed no correlation with age. The A-V difference increased with age. The age-related changes in retinal oxygen saturation are still controversial [6,13,26,27,31]. In the Indian population, it has been reported that both arterial and venous retinal oxygen saturation increase with age [27]. In a previous study on a multiethnic population [31], both arterial and venous retinal oxygen saturation decreased with age, resulting in an increase in A-V difference. Various factors including the decrease in pupil dilation, cataract formation, decrease in the vessel caliber, and atrophy of the retinal pigment epithelium or choroid may be involved in this discrepancy [24,32,33]. Judging from the results of previous reports and those of the current study, longitudinal changes in individual retinal oximetry may be small. However, these changes may be crucial in the comparisons of retinal oximetry between different generations.

One of the major limitations of our study is its cross-sectional nature. Based on a large healthy population, we investigated the age-dependent changes in retinal oximetry. However, a long follow-up study would be necessary to investigate the longitudinal changes in retinal oximetry. In addition, we excluded patients with severe cardiovascular or respiratory diseases but subjects with smoking history or systemic hypertension were included. History of controlled systemic hypertension is reported to be associated with an increased A-V difference in retinal oxygen saturation [31]. In addition, the mean age of the subjects recruited in the reproducibility test was substantially lower than that of the total study population. Subject selection may have had some influence on the measurement value and its reproducibility.

In spite of these limitations, we constructed a database of retinal oximetry for healthy Japanese. Although interphotograph repeatability was high, interevaluator and intervisit repeatability was relatively low. However, mean retinal oximetry in the 4 quadrants may contribute to higher reproducibility of measurements. Since the results of retinal oximetry show some variation across races, we might need to establish a calibration formula optimized for each race.

## Supporting Information

S1 File Minimal dataset of this study. This file shows minimal dataset of this study, formatted of Excel database.
(XLSX)
